# A Case Report on Gallbladder Agenesis: Not a Novelty but Still a Laparoscopic Surprise

**DOI:** 10.7759/cureus.20401

**Published:** 2021-12-14

**Authors:** Massimiliano Cinalli, Simone Di Russo, Paolo Panaccio, Vincenzo Casolino, Mario D'Arcangelo, Felice Mucilli, Roberto Cotellese, Federico Selvaggi

**Affiliations:** 1 Department of Medical, Oral and Biotechnological Sciences, "G. d'Annunzio" University of Chieti-Pescara, ASL2 Lanciano-Vasto-Chieti, Lanciano, ITA; 2 Unit of Surgery, Renzetti Hospital, ASL2 Lanciano-Vasto-Chieti, Lanciano, ITA; 3 Unit of Radiology, Renzetti Hospital, ASL2 Lanciano-Vasto-Chieti, Lanciano, ITA; 4 Department of Medical, Oral and Biotechnological Sciences, "G. d'Annunzio" University of Chieti-Pescara, Lanciano, ITA

**Keywords:** minimally invasive surgical procedures, diagnostic laparoscopy, transabdominal ultrasound, congenital absence of gallbladder, gallbladder agenesis

## Abstract

Gallbladder agenesis (GA) is a rare embryological anomaly that presents acute cholecystitis like-symptoms. It is often an incidental finding diagnosed during surgery. We reported a case of GA in a patient who presented with dyspepsia and acute right upper abdomen pain with ultrasonographic signs of acute lithiasic cholecystitis. The preoperative assessment, according to first-level exams, is oriented to the diagnosis of acute lithiasic cholecystitis with atrophy and sclerosis. During laparoscopy, the proximal transverse colon was found strictly adherent to gallbladder fossa. The gallbladder was found to be absent. The surgical procedure consisted of lysis of multiple colo-hepatic adhesions. The diagnosis of congenital GA was made laparoscopically. The postoperative radiological images, based on CT and MR examinations, documented the diagnosis of GA with a biliary duct anatomical variant. The recovery was uneventful and the patient remained symptom-free for more than four years. GA is a clinical challenge that still poses diagnostic and therapeutic dilemmas. Although no diagnostic and therapeutic algorithm is accepted worldwide, due to heterogeneity of clinical scenarios and the variability in hospital facilities, surgeons have to be familiar with this rare entity, and conversion in laparotomy or unnecessary operative procedures should be avoided in the same operative setting.

## Introduction

Gallbladder agenesis (GA) is a rare embryological anomaly with an incidence of 10-65 per 100,000 [1-5]. GA is associated with congenital anomalies and its occurrence is sporadic [3,4,6-8]. GA is commonly misinterpreted as acute cholecystitis with cystic duct obstruction [9]. Most patients are asymptomatic but, in almost 50% of cases, they present right upper quadrant abdominal pain, dyspepsia, nausea, and vomiting [2,10-11]. The mechanisms of biliary colic are unknown. Dysfunction of sphincter Oddi, choledocholithiasis, and biliary dyskinesia play a pivotal role and are the primary causes of pain [10]. Abdominal ultrasound (AU) is the first level examination and it has a sensitivity of 95% in diagnosing gallstones, but in cases of congenital malformations, the AU sensitivity decreases to 61% [12]. It is well known that AU is the imaging technique of choice to assess the gallbladder [12]. When AU reveals an inconclusive report, especially in the presence of a small, contracted, or shrunken gallbladder, MR cholangiogram should be combined [11,12]. No consensus is available on the appropriate management of GA and this reflects the heterogeneity of clinical scenarios and the variability of hospital facilities. For these reasons, many cases of GA have been described during the surgical procedure as an incidental finding [2-4,8,9]. Conversion to laparotomy and accurate investigation of the biliary anomaly for suspected gallbladder ectopy might be indicated although other colleagues discourage invasive and extensive surgical dissection with the aim of reducing exploration complications [13]. We describe our first case of GA, diagnosed laparoscopically, with a review of the current literature and emphasis on diagnostic and therapeutic dilemmas.

## Case presentation

**Figure 1:**
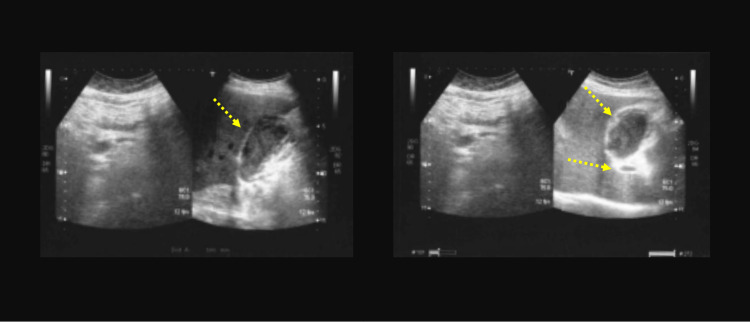
Preoperative ultrasound images mimicking acute lithiasic cholecystitis with atrophy and sclerosis. The longitudinal view of the gallbladder in the fasting state (left images, dotted arrow). The relationship of the gallbladder with the portal vein (right images, dotted arrows). The dilated loop of the bowel (right colonic flexure) is misinterpreted with acute lithiasic cholecystitis.

**Figure 2:**
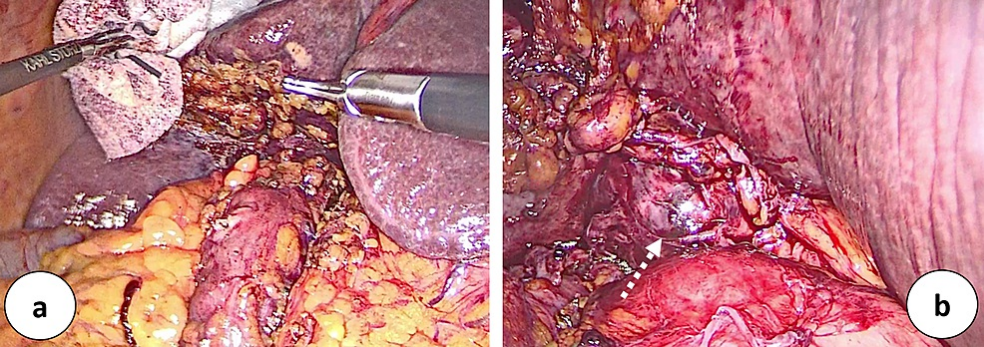
Laparoscopic view: (a) dissection of the transverse colon from the gallbladder fossa and lysis of colo-hepatic adhesions; (b) GA identified after the partial exhibition of sopraduodenal common bile duct.

**Figure 3:**
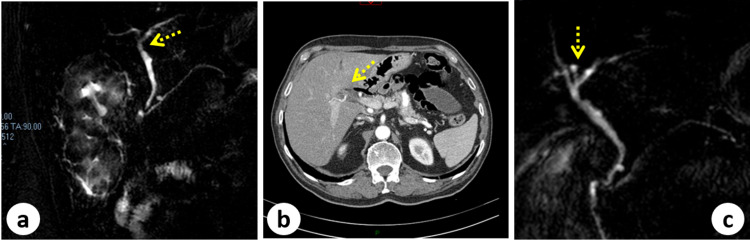
Radiological images: (a) MRCP showing GA (dotted arrow); (b) CT scan image showing right hepatic artery and its relation with right hepatic biliary duct (dotted arrow); (c) MRCP showing GA and the biliary variant of the posterior right hepatic duct with its insertion into the left hepatic duct (dotted arrow). MRCP: magnetic resonance cholangiopancreatography, GA: gallbladder agenesis

An 84-year-old-man presented acute abdominal pain localized in the right upper quadrant associated with dyspepsia and vomiting. He had no previous abdominal surgery and he was taking oral hypoglycemic agents. He had myocardial revascularization, more than 10 years ago, with aorto-coronary bypass. In his history, there were recurrent biliary colics as documented by personal examinations. Preoperative AU showed the signs of acute lithiasic cholecystitis with contracted and shrunken aspects (Figure 1). Laboratory tests documented an increased white blood cell count (15.43 × 10^3^/µl) and a total serum bilirubin level of 30.95 µmol/L (conjugated bilirubin level of 6.84 µmol/L). Liver function tests were within normal limits (AST SGOT: 19 U/L; ALT SGPT 23 U/L and GGT 54 U/L). The troponin test showed normal values (9.10 pg/ml). Laparoscopic cholecystectomy is indicated due to clinical and AU signs of acute lithiasic cholecystitis. During exploration, the proximal transverse colonic flexure was found strongly adherent to the gallbladder fossa but the gallbladder was found to be absent. The surgical minimally invasive manoeuvres consisted of lysis of multiple peritoneal adhesions and dissection of the porta hepatis (Figure 2). Unfortunately, we were unable to offer details by using intraoperative ultrasounds. Postoperatively, an abdominal CT scan and an MR cholangiogram confirmed the diagnosis of congenital GA with the presence of an intrahepatic biliary anatomical variant. This biliary anomaly consisted of the posterior right hepatic duct insertion into the left hepatic duct (Figure 3). The postoperative period was uneventful and the patient was discharged in good clinical conditions. The patient remains symptom-free for more than four years from hospital discharge.

## Discussion

First described in 1701, the pathogenesis of GA still remains unclear although an embryological malformation has been hypothesized [4-6]. GA might be an isolated finding or might be associated with other gastrointestinal, genitourinary, and cardiovascular anomalies [3,6,7]. As reported, GA is usually associated with cystic duct absence [8]. The most common symptoms are right upper abdominal pain, nausea, vomiting, and anorexia [11]. It is supposed that dysfunction of the sphincter of Oddi, choledocholithiasis, and biliary dyskinesia might play a pivotal role [10,14]. In 1988, Bennion classified GA patients into three groups: asymptomatic, symptomatic, and with multiple congenital malformations [3]. A modern classification has been drawn, dividing patients into two groups: asymptomatic and symptomatic, the latter further classified according to the presence or absence of fatal congenital malformations [9]. In clinical practice, AU is the first level examination used to study gallbladder pathologies [12]. AU has a 95% sensitivity in diagnosing gallstones but it relies on the operator's experience and on the body habitus of the patient with a sensitivity that decreases to 61% in specific conditions, especially in cases of congenital malformations [10,13]. AU is the preferred initial imaging technique, in view of its cost-effectiveness, wide availability, reduced invasiveness, and good accuracy for gallstones disease [12]. In the presence of gas artefact, inflamed periportal tissue, or subhepatic peritoneal folds, AU might lead to an uncorrected diagnosis of the shrunken or contracted gallbladder or even cholecystitis with cystic duct obstruction [13,14]. When AU reveals inconclusive reports, MR cholangiogram should be combined with AU [11]. MRCP, CT scan, and endoscopic ultrasound represent diagnostic tools with high sensitivity in GA diagnosis [15]. In our experience, the GA diagnosis is made laparoscopically and CT scan and MR cholangiogram CP are performed postoperatively (Figures 2-3). No consensus is still available on the appropriate management of GA patients during surgery [16]. Conversion to laparotomy is advocated by facilitating the exploration of a suspected ectopic gallbladder [3,16]. Some reports underline the role of intraoperative cholangiography or invasive procedure during GA management with the aim of mapping the biliary tree [9,16,17]. Other investigators, on the contrary, discourage these invasive approaches to avoid the risk of biliary complications [1]. Exploratory laparoscopy plays a diagnostic role in an emergency setting and this explains why many “laparoscopic” diagnoses of GA are reported [2,4,8,9,15,16]. Despite all this, the so-called "unnecessary operations" in GA patients are often therapeutic because after lysis of peritoneal adhesions GA patients became asymptomatic [18]. Sphincterotomy and choledocho-enteric anastomosis are alternatives for GA treatment [8]. More recently, other experiences suggest the indication of MR cholangiogram rather than unnecessary exploration when AU is inconclusive for gallbladder [11]. Conservative management with smooth muscle relaxants is suggested but in severe cases sphincterotomy or conversion to laparotomy is indicated in order to exclude ectopic gallbladder and perform bile duct exploration by using cholangiography and T-tube placement [1,8,9,16]. The problem is that it is very difficult to suspect preoperatively GA findings, especially when AU, the first level examination for biliary diseases, confirms the diagnosis of acute cholecystitis with atrophy and sclerosis. A diagnostic and therapeutic algorithm is proposed by Malde [17]. According to this algorithm, we have programmed patient care. The initial radiological investigation with AU visualized the gallbladder. For this reason, laparoscopic cholecystectomy was indicated. The pre-operative AU confirmed the diagnosis of acute lithiasic cholecystitis, but surprisingly the gallbladder was not found intraoperatively (Figures 1-2). After a laparoscopic diagnosis of GA, we prefer to abandon further surgical procedures and use postoperative radiological investigations as suggested in the decisional tree of Malde [17].

## Conclusions

In conclusion, hepato-biliary surgeons have to be familiar with this rare entity because GA therapy requires prudence. More and more frequently, GA might be a laparoscopic surprise, and the conversion to open surgery should be kept as low as possible by offering to patients all the advantages of minimally invasive procedures. A modern and accurate imaging investigations have to be indicated, whenever possible, during laparoscopy by using intraoperative ultrasounds or after the intraoperative GA finding, with the aim of avoiding biliary complications.
